# Minimally Invasive Ross Procedure in Adults, Through an Arrow-Shaped (“V” Shaped) Ministernotomy Approach

**DOI:** 10.3390/jcm15145322

**Published:** 2026-07-08

**Authors:** Ali Shadmanian, Kosha Patel, Antal Szabó-Biczók, Sándor Varga, Tamás Donauer, Szilvia Agócs, Ádám L. Balogh, Miklós Bitay

**Affiliations:** 1 Department of Cardiac Surgery, University of Szeged, 6725 Szeged, Hungary; ali.shadmanian@gmail.com (A.S.); patel.kosha@o365.u-szeged.hu (K.P.); szba01@gmail.com (A.S.-B.); svargadr@gmail.com (S.V.); gadrit@gmail.com (T.D.); 2 Department of Anesthesiology and Intensive Therapy, University of Szeged, 6725 Szeged, Hungary; agocs.sz7@gmail.com (S.A.); balogh.adam.laszlo@med.u-szeged.hu (Á.L.B.)

**Keywords:** minimally invasive, arrow-shaped ministernotomy, Ross procedure

## Abstract

**Background/Objectives:** The Ross procedure is recognized for its excellent long-term outcomes in aortic valve surgery, providing superior hemodynamic performance, freedom from anticoagulation, and a low risk of valve thrombosis. The aim of this study was to evaluate the technical feasibility and early clinical outcomes of performing the Ross procedure through a minimally invasive V-shaped (arrow-shaped) partial sternotomy in adult patients. **Methods:** Eleven consecutive adult patients underwent a Ross procedure through a 7 cm upper midline skin incision and a V-shaped (arrow-shaped) ministernotomy sternotomy extending to the third intercostal space. Cardiopulmonary bypass was established through central cannulation. The pulmonary autograft was implanted in the aortic position using the miniroot technique, followed by implantation of a pulmonary homograft in the pulmonary position. Operative, perioperative, and early echocardiographic outcomes were prospectively evaluated. **Results:** All procedures were completed successfully without conversion to full sternotomy or major intraoperative complications. The cardiopulmonary bypass and aortic cross-clamp times were consistent across the series, averaging 144 and 86 min, respectively. Intraoperative transesophageal echocardiography confirmed satisfactory function of both the autograft and homograft in all patients. Postoperative recovery was uneventful, with mechanical ventilation times ranging from 4 to 8 h and intensive care unit stays of 24–48 h. No cases of stroke, renal failure, permanent pacemaker implantation, atrial fibrillation, wound infection, sternal instability, reoperation for bleeding, readmission, or mortality occurred. Patients were discharged between postoperative days 7 and 10. At 3-month follow-up, transthoracic echocardiography demonstrated preserved left ventricular function, absence of aortic regurgitation, and low transvalvular gradients in all patients. **Conclusions:** This initial single-center case series demonstrates the technical feasibility of performing the Ross procedure through a V-shaped (arrow-shaped) partial sternotomy in carefully selected adult patients. The approach provides satisfactory operative exposure for all components of the procedure while maintaining the advantages of minimally invasive surgery. Larger studies with longer follow-up are required to further evaluate clinical outcomes and long-term durability.

## 1. Introduction

The Ross procedure [1] is renowned for its extended freedom from re-intervention and superior long-term survival rates compared to other aortic valve surgical interventions [2,3,4]. Due to these favorable outcomes, it is predominantly utilized in pediatric and young adult populations. Additionally, its excellent hemodynamic profile, avoidance of anticoagulant therapy, and minimal risk of valve thrombosis further contribute to its benefits [2,3,4]. Essentially, the Ross procedure involves the transplantation of the patient’s pulmonary autograft into the aortic position and the placement of a pulmonary homograft in the pulmonary position. By adopting a minimally invasive approach, we aim to merge the benefits of the living valve in the aortic position with those of ministernotomy, which include improved postoperative recovery, long-term quality of life, and superior cosmetic results [5]. In contrast to previous cases documented by Franke et al. in 2009 [5], our procedures were conducted through an arrow-shaped ministernotomy to the 3rd intercostal space, aligning with our standard approach for minimally invasive complex aortic surgeries, involving the aortic valve, aortic root and ascending aorta, such as the David II procedure, Yacoub procedure, Bentall procedure, cryopreserved aortic homograft implantation, and ascending aorta replacement [6] This approach provides broad operative exposure for complex aortic procedures while maintaining the advantages of minimally invasive access and is notably shorter than the “J” partial sternotomy to the 4th intercostal space. Herein, we present the initial eleven cases of Ross procedures performed via an arrow-shaped ministernotomy approach to the 3rd intercostal space in adult patients.

Although the Ross procedure provides excellent long-term survival and hemodynamic outcomes, it is traditionally performed through a full median sternotomy. Because Ross candidates are frequently young, active adults with long life expectancy, reducing surgical trauma while preserving procedural safety is particularly attractive. Minimally invasive cardiac surgery has been associated with reduced postoperative pain, lower blood loss, earlier mobilization, shorter recovery, improved cosmetic results, and greater patient satisfaction. Therefore, adapting the Ross procedure to a minimally invasive approach may combine the biological advantages of a living autograft with the benefits of limited-access cardiac surgery.

## 2. Methods

### 2.1. Patient Selection

In this retrospective study, we analyzed a total of 11 patients who underwent the Ross procedure at our institution between 2018 and 2023, via an arrow-shaped ministernotomy approach to the third intercostal space. 

The average age of the patients was 40 years, ranging from 18 to 53 years, with seven of the patients being female. All patients presented with significant aortic stenosis, and six of them also exhibited varying degrees of aortic regurgitation. Echocardiographic assessments revealed transaortic gradients exceeding 70 mmHg, alongside mild-to-severe aortic regurgitation. Importantly, all patients had preserved left ventricular function, and coronary angiography ruled out the presence of coronary artery disease. The patients’ past medical histories were unremarkable, with no chronic kidney disease, pulmonary disease, or diabetes mellitus. The body mass indexes (BMI) ranged from 20 to 31 (Table 1). Given the young age and active lifestyle of these patients, the decision was made to perform the Ross procedure using a minimally invasive approach, specifically an arrow-shaped ministernotomy approach. Patients were selected for the Ross procedure based on young age, active lifestyle, preserved ventricular function, suitability for pulmonary autograft replacement, and anticipated long-term benefit from a living valve substitute. All Ross procedures performed during the study period were undertaken through the V-shaped (arrow-shaped) ministernotomy approach; therefore, the present series represents the complete institutional Ross experience during the study interval rather than a selected subgroup.

Renal function was preserved in all patients, with no evidence of chronic kidney disease and normal estimated glomerular filtration rates. None of the patients had diabetes mellitus, chronic pulmonary disease, or significant peripheral vascular disease.

### 2.2. Operative Technique

**Figure 1:**
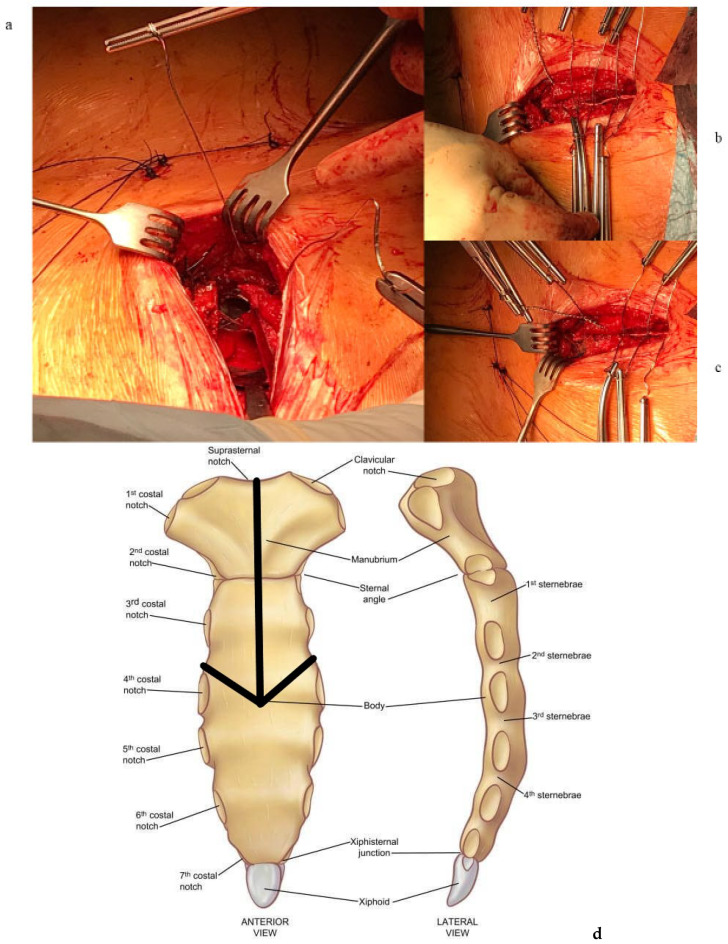
Arrow-shaped ministernotomy. (**a**). Intraoperative aspect of the longitudinal and transversal sternum division. (**b**,**c**). Closure of the arrow-shaped sternotomy. The transversal section is closed with separate wires, which are then secured in a “butterfly” fashion. The rest of the sternum is closed in the conventional way, mostly in “butterfly” fashion as well. (**d**). Schematic representation of the arrow-shaped sternotomy.

The surgical procedure began with a 6–7 cm skin incision starting from the upper end of the sternum, followed by the division of the sternum down to the third intercostal space in an arrow-shaped manner (Figure 1). This approach was chosen to provide optimal access while minimizing the invasiveness of the procedure. By dividing the sternum partially in length and transversally in both ways, left and right, we obtained a comfortable view and a large exposure for the operative field. Central cannulation for cardiopulmonary bypass (CPB) was established using a single venous cannula, which allowed for effective systemic circulation management during the operation, despite opening the right heart while harvesting the pulmonary autograft. Myocardial protection was achieved through the administration of anterograde cold crystalloid cardioplegia (Custodiol), ensuring the preservation of heart tissue during a longer procedure.

**Figure 2:**
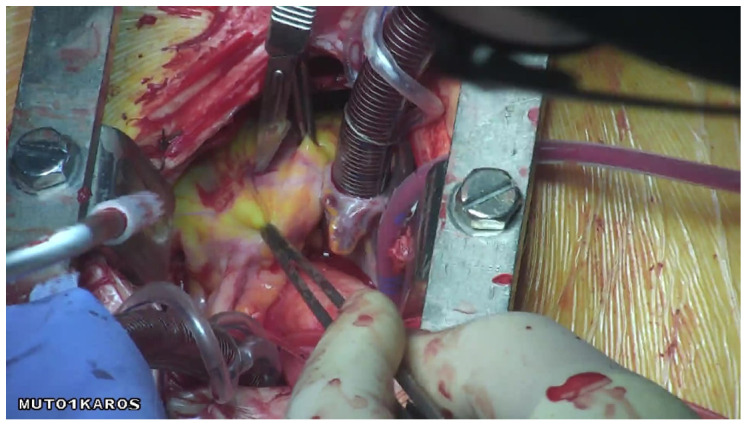
Harvesting of the pulmonary autograft, before aortic cross-clamp.

Once CPB was initiated, the pulmonary autograft was harvested before the aorta was cross-clamped (Figure 2). The diseased aortic valve was excised via a transverse aortotomy, made slightly distal to the sino-tubular junction, providing good access to the valve, coronary button and ensuring comfortable access for the implantation of the pulmonary autograft.

**Figure 3:**
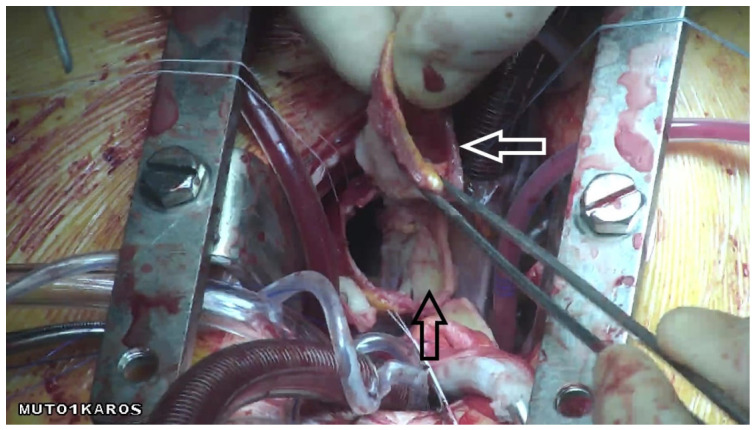
After excision of the diseased aortic valve (black arrow), the pulmonary autograft is implanted with an everted miniroot technique (white arrow).

The next step involved preparing the coronary buttons, with larger portions of the aorta containing the coronary arteries, for their re-implantation in the neo-aorta valved conduit. The diseased aortic valve was fully excised, and the pulmonary autograft was implanted into the aortic position using the everted miniroot technique (Figure 3). This technique involves securing the autograft at the annular level with three continuous 3.0 polypropylene sutures, ensuring a stable and durable valve replacement at the level of the annulus, reducing the risk of neo-aortic valve annular dilatation.

Coronary button re-implantation was performed with 5.0 continuous polypropylene sutures, first the left, then the right button, followed by the distal aortic anastomosis, which was completed using 3.0 continuous polypropylene sutures. To reduce the risk of long-term graft failure due to root dilatation, the remaining intact aortic root, particularly the noncoronary Valsalva sinus, was used to cover the corresponding portion of the autograft. Also, the above-mentioned preparation of larger coronary ostia served the same purpose.

**Figure 4:**
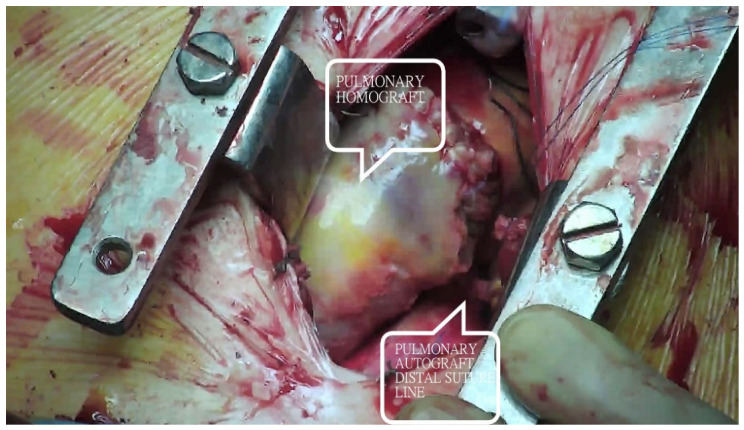
The right ventricular outflow tract is reconstructed with a pulmonary homograft. This figure shows the final result, before wound closure.

Following the successful implantation of the autograft, attention was turned to the pulmonary position. A pulmonary homograft, sized between 26 and 28 mm, was selected from the Cardiac Surgery Department’s allograft bank at the University of Szeged Biobank, Hungary. The homograft was implanted with running 3.0 polypropylene sutures at both the distal and proximal ends. The distal anastomosis was performed first to secure the homograft in place before completing the proximal anastomosis. The proximal anastomosis was performed either during aortic cross-clamp, or after declamping of the aorta, depending on the length of the aortic cross clamp time up to this step of the procedure. If the cross-clamp time proved to be long, the proximal anastomosis to the right ventricular outflow tract was performed after declamping of the aorta, without any risk of tension on the suture line, thus being as safe as by achieving it while the aorta was still cross clamped (Figure 4).

**Figure 5:**
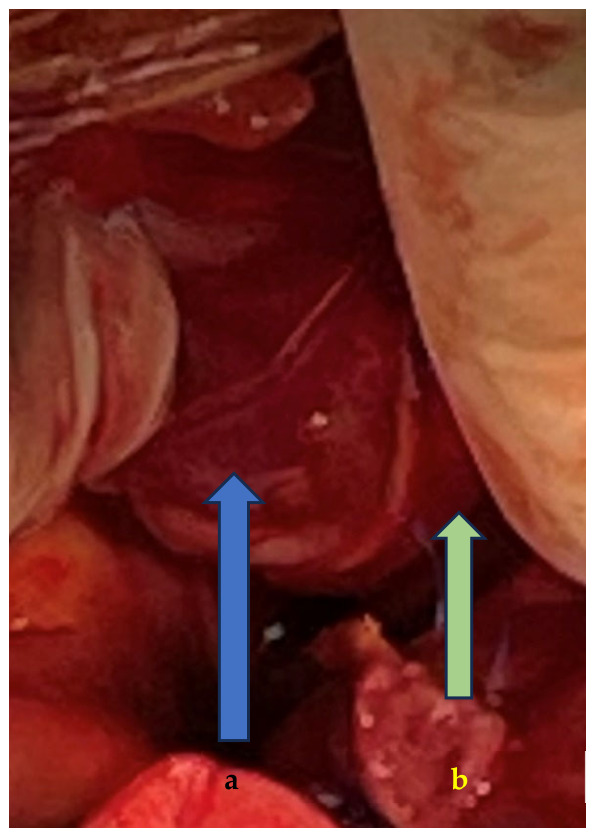
Intraoperative view of the wrapping, (**a**) blue arrow indicating the ascending aorta above the distal anastomosis and the (**b**) green arrow indicating the native aortic wall (the noncoronary sinus).

After weaning off the cardiopulmonary bypass, decannulation and hemostasis control was achieved pharmacologically and surgically, if necessary. This was followed by the wrapping of the pulmonary autograft with the native aortic wall, consisting mainly of the noncoronary Valsalva sinus (Figure 5), to prevent dilatation of the pulmonary autograft in the future.

**Figure 6:**
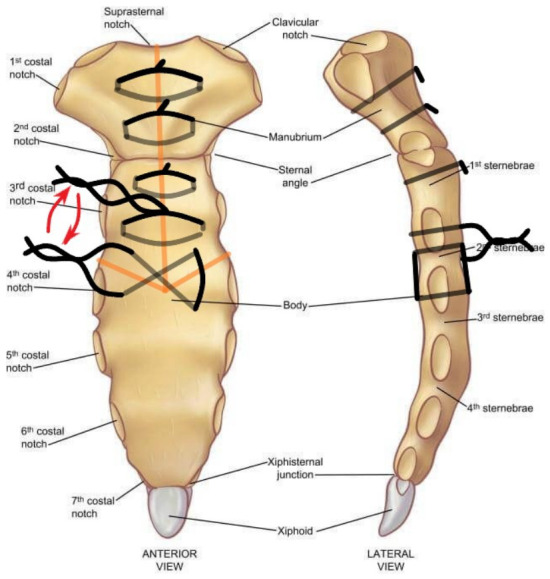
Schematic drawing of the closure of the arrow-shaped ministernotomy.

Finally, the sternum was closed in a manner that achieves maximum long-term stability (Figure 1b,c; Figure 6).

This operative approach, which emphasizes minimal invasiveness while ensuring robust surgical outcomes, was employed across all 11 cases, with good reproducibility.

## 3. Results

The aortic cross-clamp and perfusion times were consistent, ranging from 84 to 90 min and 144 to 184 min, respectively. Intraoperative transesophageal echocardiography confirmed satisfactory function of both the autograft and homograft valves, with no signs of regurgitation or transvalvular gradient. The postoperative period was uneventful, with patients being extubated on the same day as the surgery, typically 4 to 6 h after arriving in the intensive care unit (ICU). Postoperative drainage ranged between 200 and 700 mL, and none of the patients required reoperation for bleeding. The need for red blood cell transfusions was between 2 and 5 units. No atrioventricular block occurred postoperatively, eliminating the need for pacemaker implantation, and there were no cases of renal failure (Table 1). Patients were transferred to the ward the next morning. Most were discharged between postoperative days 7 and 10, except for one patient who tested positive for COVID-19 (SARS-CoV-2) after the procedure but remained asymptomatic (Table 1).

Pre-discharge evaluations, including chest X-ray, electrocardiogram (ECG), and transthoracic echocardiography (TTE), showed excellent results. There were no pleural or pericardial effusions, and both the neo-aortic and pulmonary valves were functioning well, alongside good left ventricular function. These outcomes were consistent with findings at the 3-month postoperative follow-up.

At the 3-month follow-up, transthoracic echocardiography demonstrated preserved left ventricular systolic function in all patients, with ejection fractions ranging from 55% to 68%. No patient exhibited aortic regurgitation, and all neo-aortic valves demonstrated excellent hemodynamic performance, with mean transvalvular gradients ranging from 3 to 8 mmHg. All patients remained in sinus rhythm. No evidence of autograft dysfunction, pulmonary homograft dysfunction, or need for reintervention was observed during the follow-up period (Table 2).

Early postoperative recovery was uneventful in all patients. Mechanical ventilation time ranged between 4 and 8 h, while intensive care unit stay ranged from 24 to 48 h. No patient required reoperation for bleeding, permanent pacemaker implantation, renal replacement therapy, reintubation, or readmission. No episodes of postoperative atrial fibrillation, wound infection, sternal instability, stroke, or mortality were observed during the early postoperative period (Table 3).

## 4. Discussion

The Ross procedure [1] has long been recognized as one of the most effective methods for aortic valve replacement, particularly in pediatric and young adult populations. This procedure is favored for its excellent long-term outcomes, with substantial data supporting its effectiveness across various age groups, from children to young adults, and even up to patients aged 55–60 [2,3]. One of the primary reasons for the Ross procedure’s success lies in the exceptional hemodynamic performance of the pulmonary autograft, which is used to replace the diseased aortic valve. Additionally, the procedure eliminates the need for lifelong anticoagulant therapy, making it an attractive option for young, active individuals who want to avoid the limitations and risks associated with anticoagulation, such as bleeding complications and the need for regular monitoring.

Given these advantages, we sought to explore whether the Ross procedure could be further optimized by employing a minimally invasive approach, specifically a ministernotomy to the 3rd intercostal space. This approach, performed in an arrow-shaped fashion, is already used routinely in our institution for various complex aortic procedures, such as the David II, Yacoub, and Bentall procedures, cryopreserved aortic homograft implantation, and ascending aorta replacement [7,8]. This technique, illustrated in Figure 1, offers several advantages over traditional full sternotomy, especially for young, active patients who benefit from a less invasive procedure. The V-shaped approach was selected because it reproduces the operative exposure routinely used in our minimally invasive complex aortic surgery program. In our experience, it provides excellent visualization for pulmonary autograft harvest, aortic root reconstruction, coronary button reimplantation, and pulmonary homograft implantation. We believe that the bilateral extension of the V-shaped sternotomy provides broader exposure than an L-shaped approach, which may be advantageous during the multiple reconstructive stages of the Ross procedure. However, the present study was not designed to compare minimally invasive access strategies directly, and therefore no definitive conclusions regarding superiority can be drawn. The ministernotomy approach may offer potential advantages regarding postoperative recovery and cosmetic appearance, although these outcomes were not formally evaluated in the present study [6,9].

In the case of the arrow-shaped ministernotomy, by dividing the sternum transversally in both directions, this approach allows for a wider operative field than other minimal access techniques while still reducing the length of the sternotomy. The broader operative exposure may facilitate performance of the multiple reconstructive stages of the Ross procedure while preserving the minimally invasive nature of the approach. According to Idserd et al., symmetrical bilateral retraction during V-shaped ministernotomy may reduce tension compared to one side (right) retraction on the internal thoracic arteries and has been proposed as one potential contributor to favorable postoperative recovery. However, the present study was not designed to evaluate this mechanism directly [6]. Although no wound infections or sternal wound complications occurred in the present series, perioperative infection prevention remains an important component of care in all cardiac surgical procedures, including minimally invasive sternotomy approaches. All patients were managed according to our institution’s standard perioperative infection-prevention protocol. Additionally, the reduced length of the sternotomy results in better cosmetic outcomes, an important consideration for young and active patients concerned about visible scarring. Importantly, the durations of cardiopulmonary bypass (CPB) and aortic cross-clamping during the Ross procedures performed with this approach were within acceptable limits and were within acceptable ranges for Ross procedures reported in the literature. This consistency in surgical times is a key finding, as it demonstrates that this type of minimally invasive approach does not compromise the safety or reproducibility of the Ross procedure, which is known to be a technically demanding operation.

The Ross procedure itself presents significant challenges, largely due to the complexities involved in autograft and homograft implantation. Therefore, the importance of providing the surgeon with a generous and comfortable operative field, even in a minimally invasive setting, cannot be overstated. The arrow-shaped sternotomy, despite its minimally invasive nature, offers excellent exposure to the heart and great vessels, significantly improving the surgeon’s comfort to perform this rather demanding procedure. This may facilitate technical performance of the procedure, reduce the risk of complications, and contribute to better overall patient outcomes.

Based on our institutional experience spanning approximately 20 years, the V-shaped (arrow-shaped) ministernotomy has been routinely used for a variety of complex aortic procedures, including isolated aortic valve replacement, valve-sparing root replacement, ascending aortic replacement, and combined procedures. During this period, only three cases of sternal instability were observed, all associated with deep sternal wound infection. Although the present Ross series is too small to permit formal comparison with full sternotomy or other minimally invasive approaches, these observations suggest that satisfactory long-term sternal stability can be achieved with the closure technique described herein.

One of the recognized limitations of the Ross procedure is the potential for late failure of the pulmonary autograft. This is often caused by the dilatation of the neo-aortic root and the sino-tubular junction, which can lead to valve dysfunction over time. To prevent this complication, various techniques have been developed, including the use of Dacron grafts to wrap the pulmonary autograft and provide additional structural support [10]. However, in our series of patients, we employed a different strategy to reinforce the neo-aortic root. Instead of using a synthetic graft, we wrapped the neo-aortic root with the patient’s native aortic wall, specifically utilizing the noncoronary sinus of Valsalva Figure 6. This approach has several advantages over traditional Dacron wrapping. First, it provides a certain degree of flexibility to the aortic root, preserving its natural bulb shape, which is crucial for the long-term function of the neo-aortic valve. Research has shown that maintaining this natural shape is essential for preventing valve dysfunction and ensuring the longevity of the autograft.

In addition to wrapping the neo-aortic root, we prepared larger coronary buttons during the procedure, which further stabilized the pulmonary autograft. This enhanced stability helps to reduce the risk of dilatation and provides additional support to the autograft, thus increasing its durability. Our approach, by preserving the flexibility and natural shape of the aortic root, offers a distinct advantage over the more rigid support provided by synthetic grafts, which can sometimes lead to complications such as aneurysm formation or valve dysfunction over time.

One of the criticisms frequently leveled against the Ross procedure is the fact that the pulmonary valve, which is used to replace the aortic valve, is itself replaced with a pulmonary homograft. Pulmonary homografts, while effective in the short and medium term, are known to degenerate over time, leading to the need for reoperation in some cases. This degeneration is often attributed to immune reactions, including an acute immune response to the homograft. The homografts used in the Ross procedure are typically selected based on size alone, without regard to ABO blood group compatibility or HLA matching, which may contribute to the immune response and subsequent degeneration [11].

The following observations derive from previous institutional experience and are presented as background information relevant to pulmonary homograft durability rather than as results of the current cohort. To mitigate the risk of early homograft degeneration, we implemented a postoperative protocol involving the use of steroid therapy. Our patients received prednisolone at doses ranging from 4 to 12 mg for the first postoperative week. This steroid therapy is designed to prevent acute rejection by reducing the immune response during the critical early phase after homograft implantation. Additionally, we hypothesize that the process of homograft decellularization after implantation, combined with the reduced immune response from steroid therapy, may facilitate in vivo recellularization of the homograft with host endothelial cells. This process could extend the longevity of the pulmonary homograft by making it more like a native tissue rather than a foreign graft.

This hypothesis is supported by findings from DNA testing of explanted homograft valves. In two of our previous modified subcoronary aortic homograft implantation cases, we performed a “paternity” DNA test on cells from the explanted homograft valves, comparing them to cells from the patient’s harvested pericardial tissue. The results confirmed that the endothelial cells on the homograft were genetically identical to the patient’s own cells, providing evidence of in vivo recellularization. Additionally, these explanted valves were free from calcification or fibrosis and retained their normal elasticity, suggesting that the recellularization process may indeed prolong the life of the homograft.

Patient selection remains critical for the successful application of this technique. In our opinion, the arrow-shaped ministernotomy Ross procedure is particularly suitable for young and middle-aged adults with isolated aortic valve disease who are expected to derive long-term benefit from the Ross procedure and who value rapid recovery and improved cosmetic outcomes.

### Limitations

This study represents a single-center case series with a limited number of patients and relatively short follow-up. Consequently, conclusions regarding long-term durability, comparative safety, effectiveness, or superiority over alternative minimally invasive approaches cannot be established. Furthermore, no direct comparison was performed with full sternotomy, J-shaped ministernotomy, or L-shaped ministernotomy. Larger multicenter studies with longer follow-up will be required to further evaluate clinical outcomes and validate the reproducibility of this approach.

## 5. Conclusions

The results from this initial case series demonstrate that the Ross procedure can be performed through a V-shaped (arrow-shaped) ministernotomy in carefully selected adult patients. The approach appears technically feasible and provides satisfactory operative exposure for all components of the procedure. However, larger studies with longer follow-up are required before conclusions regarding safety, effectiveness, or superiority over other surgical approaches can be established. The uneventful postoperative course, characterized by a brief one-day stay in the intensive care unit (ICU) and a seven- to ten-day hospitalization period, further supports the feasibility of this technique.

While early steroid therapy may potentially prolong the longevity of the pulmonary homograft, further research is required to confirm these findings and establish more definitive protocols. Nonetheless, our initial results are promising and suggest that this approach may represent an additional surgical strategy for managing complex aortic valve disease in young and active patients, but further investigation in larger cohorts with longer follow-up is warranted.

## Figures and Tables

**Table 1:** Patient characteristics pre- and postoperatively.

Patient Number /Sex	Age at the Operation	Preoperative EF%	Preoperative ECG	Postoperative EF%	Postoperative ECG	BMI	Preoperative Valve Pathology AS/AI	CKD	PHT	DM	eGFR (mL/min/1.73 m^2^)	Mean/Peak Gradient (mmHg)
1/F	42	67%	SR	76%	SR	21	+/−	None	None	None	83	48/95
2/M	47	62%	SR	60%	SR	23	+/+	None	None	None	92	51/105
3/M	36	66%	SR	56%	SR	20	+/−	None	None	None	89	46/88
4/F	46	56%	SR	68%	SR	28	+/−	None	None	None	93	56/124
5/F	50	66	SR	65%	SR	25	+/+	None	None	None	95	51/91
6/F	51	70	SR	68%	SR	23	+/+	None	None	None	88	44/86
7/M	26	59	SR	65%	SR	31	+/−	None	None	None	87	57/117
8/F	18	63	SR	60%	SR	24	+/+	None	None	None	81	49/92
9/F	53	62	SR	55%	SR	26	+/−	None	None	None	94	49/101
10/F	44	45	SR	51%	SR	24	+/+	None	None	None	89	47/92
11/M	30	61	SR	48%	SR	21	+/+	None	None	None	93	49/99

BMI = body mass index, AS/AI = aortic stenosis/aortic regurgitation, CKD = chronic kidney disease, PHT = pulmonary hypertension, DM = diabetes mellitus, EF = ejection fraction, SR = sinus rhythm, eGFR = estimated glomerular filtration rate, + = present, − = absent.

**Table 2:** Three-month transthoracic echocardiographic follow-up results. (SR = sinus rhythm).

Patient	EF (%)	AR	Mean Neo-Aortic Gradient (mmHg)	Rhythm
1	68	None	6	SR
2	60	None	8	SR
3	58	None	4	SR
4	65	None	5	SR
5	63	None	7	SR
6	65	None	3	SR
7	66	None	7	SR
8	62	None	3	SR
9	58	None	6	SR
10	55	None	3	SR
11	55	None	5	SR

**Table 3:** Early perioperative outcomes.

Result	Variable
Cardiopulmonary bypass time	144 min (mean)
Aortic cross-clamp time	86 min (mean)
Mechanical ventilation time	4–8 h
ICU stay	24–48 h
Reoperation for bleeding	0
Permanent pacemaker implantation	0
Renal failure	0
Stroke	0
Wound infection	0
Sternal instability	0
Reintubation	0
Atrial fibrillation	0
Readmission	0
Mortality	0

## Data Availability

The datasets generated and/or analyzed during the current study are not publicly available due to institutional regulations and patient confidentiality requirements but are available from the corresponding author upon reasonable request and subject to approval by the Institutional Review Board of the University of Szeged.
